# Conservation and divergence of known apicomplexan transcriptional regulons

**DOI:** 10.1186/1471-2164-11-147

**Published:** 2010-03-03

**Authors:** Kobby Essien, Christian J Stoeckert

**Affiliations:** 1 Department of Bioengineering, University of Pennsylvania, 240 Skirkanich Hall, 210 South 33rd Street, Philadelphia, Pennsylvania 19104, USA; 2 Center for Bioinformatics, University of Pennsylvania, 1420 Blockley Hall, 423 Guardian Drive, Philadelphia, Pennsylvania 19104, USA; 3 Department of Genetics, School of Medicine, University of Pennsylvania, 415 Curie Boulevard, Philadelphia, Pennsylvania 19104, USA

## Abstract

**Background:**

The apicomplexans are a diverse phylum of parasites causing an assortment of diseases including malaria in a wide variety of animals and lymphoproliferation in cattle. Little is known about how these varied parasites regulate their transcriptional regulons. Even less is known about how regulon systems, consisting of transcription factors and target genes together with their associated biological process, evolve in these diverse parasites.

**Results:**

In order to obtain insights into the differences in transcriptional regulation between these parasites we compared the orthology profiles of putative malaria transcription factors across species and examined the enrichment patterns of four binding sites across eleven apicomplexans.

About three-fifths of the factors are broadly conserved in several phylogenetic orders of sequenced apicomplexans. This observation suggests the existence of regulons whose regulation is conserved across this ancient phylum. Transcription factors not broadly conserved across the phylum are possibly involved in regulon systems that have diverged between species. Examining binding site enrichment patterns in light of transcription factor conservation patterns suggests a second mode via which regulon systems may diverge - rewiring of existing transcription factors and their associated binding sites in specific ways. Integrating binding sites with transcription factor conservation patterns also facilitated prediction of putative regulators for one of the binding sites.

**Conclusions:**

Even though transcription factors are underrepresented in apicomplexans, the distribution of these factors and their associated regulons reflect common and family-specific transcriptional regulatory processes.

## Background

The apicomplexans are a phylum of about 5000 protozoan organisms that parasitize almost all animals [1]. Despite sharing three invasion-related organelles, collectively called the apical complex, these organisms are diverse and pursue a wide variety of lifestyles that result in varied diseases of medical and veterinary importance. *Plasmodium *species are malaria-causing mosquito-borne parasites. *Theileria *species are tick-transmitted parasites that cause lymphoproliferative diseases in cattle. *Cryptosporidium *species do not require a vector and are spread from host-to-host causing cryptosporidiosis, an acute diarrheal disease in mammals.

Sequencing and subsequent analysis of the human malaria parasite *Plasmodium falciparum *revealed that the parasite has few known or putative transcription factors [2,3]. A similar dearth of transcription factors has been observed in other apicomplexans [4,5]. Even with the discovery of the existence of a family of about 26 AP2 transcription factors in these parasites [6], compared to other single-celled eukaryotes such as yeast, apicomplexans still have a paucity of DNA-binding transcription factors.

In their analysis of *Plasmodium *noncoding regions, Imamura and colleagues identified three putative binding sites in rodent parasites [7]. We recently, described seven putative transcription factor binding sites that were conserved in at least two *Plasmodium *species [5]. Three of these binding sites were broadly conserved across human, primate and rodent malaria parasites. Other work has described the conservation of binding sites across *Theileria *[8] and *Cryptosporidium *species [9]. These results have raised the question of how broadly conserved the control of transcription is across apicomplexans. Specifically, is transcriptional control largely conserved across apicomplexans or are transcription factors and their binding sites in promoters of target genes only conserved between closely related subsets of apicomplexans?

Differences in transcriptional control are thought to contribute to species diversity [10]. High throughput genomics approaches have highlighted divergence of known binding sites of conserved transcription factors in related yeast species [11] and between human and mouse [12]. It has been noted that the *P. falciparum *AP2 transcription factor PF14_0633 and its *Cryptosporidium parvum *ortholog, cgd2_3490, likely regulate different sets of genes [13]. The nucleotide word TGCATGCA, identical to the PF14_0633 binding site, is overrepresented in all sequenced apicomplexan genomes except *Theileria *[14,15]. This suggests that the diversification principles at work in apicomplexa may be similar to those at work in other eukaryotes.

Despite belonging to the same phylum, apicomplexans are characterized by a striking amount of diversity. Members of the blood-invading haemosporidian order of parasites include the mosquito-transmitted *Plasmodium *species that cause malaria. Even though *Theileria *species also invade red blood cells, they belong to the piroplasmid order of apicomplexans and are transmitted by ticks and mostly cause lymphoproliferative diseases in cattle. Then there are *Toxoplasma *and *Neospora *both of which fall in the eucoccidioridian order and do not require a host and exhibit very little cell tropism. The issue of whether these parasites modulate their transcriptional control to pursue such extremely diverse lifestyles and inhabit distinct niches remains largely unexplored.

In this work, we examined the conservation of putative *Plasmodium falciparum *transcription factors across apicomplexans. We discover that disparate subsets of apicomplexans likely have different sets of transcription factors suggesting divergence in the complements of factors present in these species. Additionally, our analysis reveals that about three-fifths of putative *Plasmodium falciparum *transcription factors have an ortholog in at least one non-*Plasmodium *apicomplexan. Enrichment analysis of previously identified promoter binding sites across species also suggests large-scale differences in transcriptional control in disparate apicomplexans. None of four transcriptional regulons considered in detail were conserved across all apicomplexa. However, the conservation of two regulons spanned blood-invading phylogenetic orders within the phylum. By matching the conservation profiles of putative transcription factors with the enrichment profiles of binding sites we were able to predict candidate transcription factors for a previously partially characterized ribosomal regulon. Finally, by integrating the conservation analysis of transcription factors with the enrichment analysis of binding sites we detected two possible mechanisms via which regulons may be modified in these parasites.

## Results

In its simplest form, a transcriptional system for a regulon can be reduced to three components: a transcription factor, the set of target genes or regulon identifiable by an upstream binding site that is bound by the transcription factor, and the biological process connected to the regulon. We pursue complementary approaches to assess whether regulons differ between apicomplexans. The first approach entails determining whether transcription factors from the most widely studied apicomplexan, *P. falciparum*, are conserved in other apicomplexans. In the second approach, we seek to determine whether four previously experimentally characterized transcription factor binding sites from *P. falciparum *and *P. berghei *are conserved (enriched) upstream of similar genes in different parasites.

### Conservation patterns of putative *P. falciparum *transcription factors

**Figure 1:**
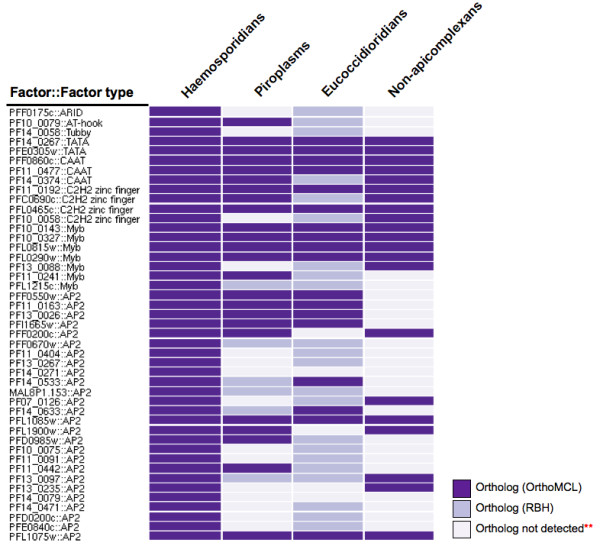
**Conservation patterns of putative *Plasmodium falciparum *transcription factors**. Orthologs predicted via reciprocal best BLAST hits (RBH) are indicated with light blue and those predicted by OrthoMCL are indicated with purple. 42.24% of the transcription factors do not have orthologs in non-*Plasmodium *apicomplexans demonstrating order-level specificity of transcriptional networks. ** The figure does not incorporate RBH information for non-apicomplexans. It does however include the associated orthology information for OrthoMCL.

The well-known diversity of lifestyles of disparate apicomplexans as well as the age of the phylum (700-900 million years [16]) led us to hypothesize that significant differences exist in the sets of transcription factors present in each species. To test this hypothesis we generated a list of putative *P. falciparum *transcription factors by identifying all *P. falciparum *proteins containing known DNA-binding domains and examined the conservation patterns of these proteins across three disparate sets of apicomplexans (Figure 1). We limit ourselves to domains possibly involved in sequence-specific DNA-binding, consequently our list of predicted *P. falciparum *transcription factors (Additional File 1) is smaller than the list of transcription associated proteins assembled by Coulson and colleagues in their comparison of transcriptional control between *P. falciparum *and other eukaryotes [3].

To establish true orthology between two genes, one would have to perform a phylogenetic study and demonstrate that a pair of genes descended from a common ancestor. This would be followed up with biological validation that the two genes do in fact have the same function in each of the two species. However detailed studies of that nature are not available for most apicomplexan genes. Consequently, we considered putative orthologs established by two commonly utilized methods i) orthology prediction via reciprocal best BLAST hits (RBH) between species and ii) orthology prediction obtained from OrthoMCL, which couples reciprocal best BLAST hits with protein clustering and offers additional refinements to orthology prediction including detecting co-orthologs and accounting for genome-wide base composition biases [17].

Initial analyses using the reciprocal best BLAST hits (RBH) method for ortholog identification revealed that not all transcription factors are conserved across the apicomplexan phylum (Figure 1). In order to make stronger claims about the conservation (or lack thereof) of transcription factors, we reran the analyses using data from OrthoMCL. In all subsequent analyses, we rely on OrthoMCL for ortholog predictions because of the aforementioned advantages that OrthoMCL has over the reciprocal best BLAST hits approach.

Analysis of putative transcription factors by OrthoMCL revealed that essentially all *P. falciparum *predicted transcription factors have orthologs in at least one other *Plasmodium *species (haemosporidians) (Figure 1). However, only 57.76% of *P. falciparum *predicted factors have an ortholog in at least one non-*Plasmodium *apicomplexan (piroplasms and eucoccidioridians). To determine whether transcription factors from other apicomplexans also have similar numbers of orthologs in *P. falciparum*, we predicted transcription factors in other apicomplexans using the methodology used for predicting *P. falciparum *transcription factors. We then checked for the presence of orthologs of these factors in *P. falciparum*. On average, 94.13% of primate-infecting *P. vivax *and *P. knowlesi *factors are conserved in *P. falciparum*, as are 78.91% of the factors from the rodent malaria parasites *P. berghei *and *P. yoelii*. The piroplasms *T. annulata *and *T. parva *have on average 33.44% of their predicted factors present in *P. falciparum *while the eucoccidioridians *T. gondii *and *N. caninum *have only 15.42%. Finally, *C. parvum *has 28.57% of its predicted factors present in *P. falciparum*. Lists of the predicted transcription factors in the other species are presented in Additional File 1.

The level of conservation of transcription factors between *P. falciparum *and the non-*Plasmodium *species was surprising given the age of the phylum. In an examination of fungus-specific predicted transcriptional regulators it was noted that the budding yeast *Saccharomyces cerevisiae *and the fission yeast *Schizosaccharomyces pombe*, which are separated by 400-600 million years of evolution [18], share only 15% of fungal-specific regulators [19]. While we cannot directly compare data from the aforementioned yeast study to ours as different criteria for factor prediction were utilized, we did not expect to see extremely high levels of conservation among the parasite species under consideration as they belong to a phylum estimated to be 700-900 million years old [16].

### Assessing conservation of *P. falciparum *merozoite invasion, ookinete stage, ribosomal gene, and sporozoite stage regulons across apicomplexan species

**Figure 2:**
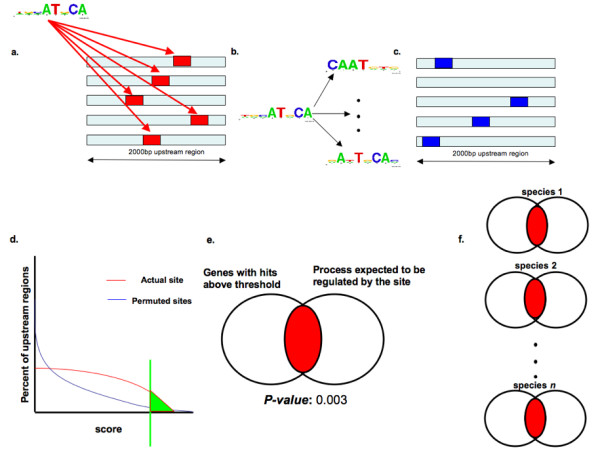
**Assessing conservation (enrichment) of binding sites across species**. a. The best instance of the binding site being studied (one of the four in Figure 3) is identified upstream of each gene in the genome. b. The binding site is then permuted 100 times to generate 100 random sites with the same base composition as the real binding site. c. The best instance of each permuted site in the regions upstream of each gene is located in the same genome. d. The distribution of scores for the real site is compared to that of the permuted sites and score at the top one percentile of permuted sites is used as a cutoff for identifying high confidence hits for the real site. e. A *p-value *is computed representing the statistical significance of the overlap between genes whose upstream regions have hits for the real site and the list of genes in the process expected to be regulated by the site. f. The enrichment analysis (steps a-e) is repeated in all genomes and enrichment *p-values *are compared across species.

We further tested the hypothesis that transcriptional regulons differ significantly between apicomplexans by examining whether transcription factor binding sites were conserved (enriched) upstream of the same sets of genes in eleven apicomplexan species. Hits or targets of a transcription factor were identified as those genes with a high confidence instance of the associated binding site in their 2000 bp upstream region (for details see Figure 2 and Methods).

**Figure 3:**
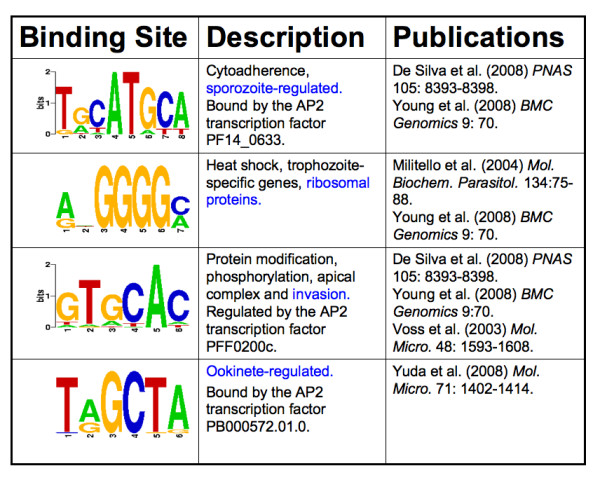
**Transcription factor binding sites considered in this study**. The functional contexts considered in the study are highlighted in blue. The transcription factor for the site, if known, and key publications are also presented.

We examined the conservation of regulons associated with four binding sites in particular (Figure 3). We focus on these sites as they are well described in the literature. De Silva and colleagues used protein binding microarrays (PBM) to show that the first and third sites in Figure 3 are bound by the AP2 transcription factor PF14_0633 [13,20] and PFF0200c [13] respectively (the functionality of the third site had been previously established by Voss et al. [21]). The transcription factor binding to the second site is unknown but the functionality of the site has been determined via deletion assays by Militello et al. [22]. Using chromatin immunoprecipitation (ChIP) and electromobility shift assays (EMSA), Yuda et al. [23] recently confirmed PF-O (PB000572.01.0) as the transcription factor for the fourth site.

For each of the four binding sites, a regulon was identified as conserved in a species if its known experimentally-associated transcription factor (See Figure 3) was present (as an ortholog) in the species and hits for the site have statistically significant overlap (enrichment) with the set of genes expected to be regulated by the site (expected regulon).

### Cell invasion regulon

**Figure 4:**
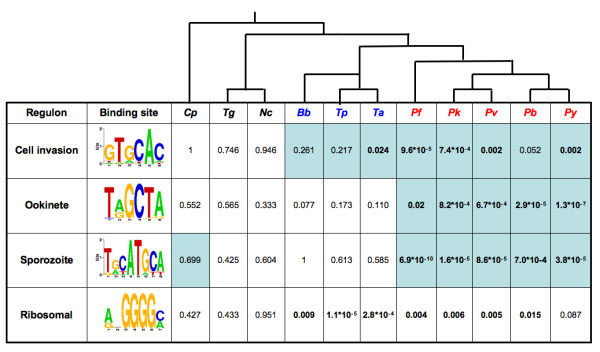
**Conservation of the four regulons**. Regulons have generally diverged across the apicomplexan phylum but genera-related conservation patterns are evident. This table presents enrichment *p-values *for each of the four regulons (rows) in each of the eleven species studied (columns). Species names (column headings) are abbreviated as presented in Table 1. Significant enrichment *p-values *are given in boldface. If the known factor for a particular regulon is conserved in a species (as determined in earlier analysis of conservation of patterns of putative transcription factors) we highlight the cell for that entry in light blue.

Conservation of enrichment of the merozoite cell invasion binding site in *Plasmodium *species and *T. annulata *only suggests a divergence in this system among members of the phylum (Figure 4). Curiously, the cell invasion binding site was not conserved in *T. parva *and *B. bovis*. Generally, the trend of *p-values *suggests a greater extent of enrichment in *T. parva *and *B. bovis *than in *T. gondii, N. caninum *and *C. parvum*.

We examined the conservation pattern of the cell invasion site's known regulator across sequenced apicomplexans. The AP2 transcription factor PFF0200c had orthologs in *Plasmodium *species, the two *Theilerias *and *Babesia *suggesting that the factor is conserved across the aforementioned species (Figures 1 and 4). Conservation of the factor in all the piroplasms with enrichment for the site in only one of the three piroplasms suggests partial conservation of the regulon in piroplasms. The lack of significant enrichment *p-values *and absence of PFF0200c orthologs in *T. gondii, N. caninum *and *C. parvum *suggests that this regulon is not conserved in eucoccidioridians.

### Ookinete regulon

The ookinete is an invasive stage of *Plasmodium *parasites within the mosquito. Enrichment for the ookinete binding site is seen only in *Plasmodium *species. The orthology analysis suggested that the factor is conserved in all *Plasmodium *species (Figure 4).

### Ribosomal regulon

The G-box binding site is not enriched upstream of ribosomal genes across all sequenced apicomplexans. This binding site is generally conserved upstream of ribosomal genes in *Plasmodium *species, the two *Theilerias *and *B. bovis *(Figure 4). As in the case of the cell invasion and ookinete sites, this ribosomal binding site is not conserved in *T. gondii, N. caninum *and *C. parvum*.

The transcription factor associated with the G-box has not been identified so its orthology pattern could not be assessed. As seen in Figure 1, eight putative transcription factors have similar orthology patterns as the G-box - absent in all the three eucoccidioridians and present in some haemosporidians and some piroplasms. Any of these eight proteins (Additional File 2) may be negative or positive regulators of G-box associated ribosomal genes. One of the eight the proteins, AP2 protein PFF0200c, was ruled out as a regulator for these genes as it binds to the cell invasion site identified by De Silva et al. [13] and is also not enriched upstream of ribosomal genes in any of the species considered (Table S6 in Additional File 3). Additionally, another of the eight proteins is predicted to be a CAAT-binding factor (Additional File 2) consequently, it too is unlikely to bind to the G-box.

**Figure 5:**
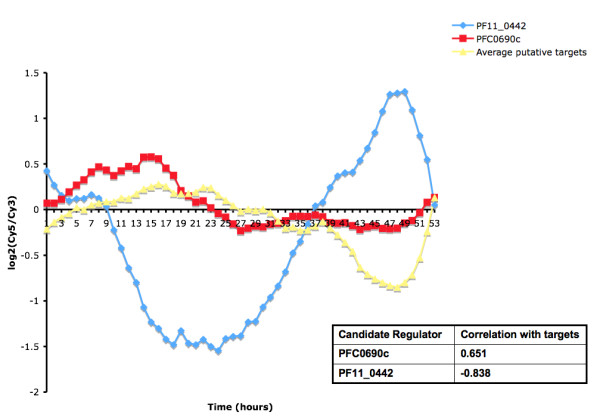
**Comparison of the expression of two putative G-box regulators and average expression of putative targets**. The highest-ranking candidate positive regulator, PFC0690c, has a correlation of 0.651 with the set of ribosomal genes with high confidence G-box hits (n = 11). The highest-ranking negative regulator, PF11_0442, has a correlation of -0.838 with these same genes. In both cases, the expression of these putative targets lags that of the candidate regulators.

To further explore the possibility that the remaining six proteins regulate the ribosomal genes in question, we compared the expression patterns of these candidate regulators to those of ribosomal genes with G-box hits (these high confidence putative G-box hits are presented in Additional File 4). Of these six, two are positively correlated with the average profile of G-box associated ribosomal genes and the other four are negatively correlated (Additional File 2). The protein with the highest positive correlation (r = 0.651) and hence the highest-ranking candidate positive regulator is the C2H2 zinc finger protein PFC0690c. The protein with the highest negative correlation (r = -0.838) and hence the highest-ranking candidate negative regulator is the AP2 protein PF11_0442. Figure 5 shows that the average profile of the G-box associated ribosomal proteins lags the profiles of both these high-ranking candidate regulators.

### Sporozoite regulon

This regulon is conserved only in *Plasmodium *species suggesting that its role in these species is quite specific (Figure 4). Its associated regulator PF14_0633 has orthologs in all other *Plasmodium *species and *C. Parvum*.

### A putative model of ribosomal gene regulation in apicomplexans

**Figure 6:**
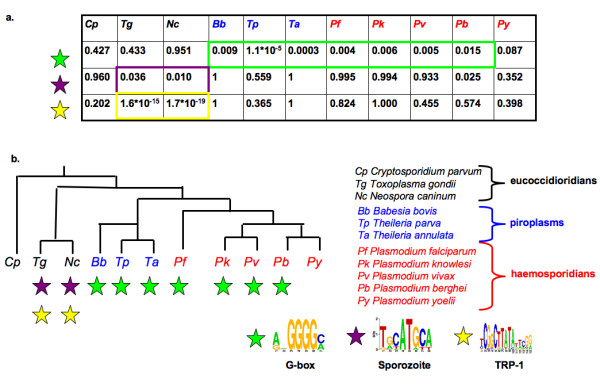
**The ribosomal regulon system in apicomplexans**. a) Enrichment *p-values *of three binding sites upstream of ribosomal genes. Stars are color-coded to represent the three transcription factor binding sites at the bottom of the image. The G-box, sporozoite and TRP-1 sites are 'starred' with green, purple and yellow respectively. b) Schematic phylogeny of apicomplexan species considered. Stars below a particular species indicate that the corresponding binding site likely regulates ribosomal genes in that particular species.

In our earlier analysis of ribosomal genes, we observed that the G-box was not enriched upstream of *T. gondii*, *N. caninum *and *C. parvum *ribosomal genes. The binding site associated with *P. falciparum *sporozoite genes has been independently implicated in the regulation of *T. gondii *ribosomal genes [24]. We tested the hypothesis that this same binding site is enriched upstream of ribosomal genes in *N. caninum*, which is closely related to *T. gondii*, and to *C. parvum *which though separate from all the other apicomplexans considered is classed together with *T. gondii *and *N. caninum *as an eucoccidioridian. This hypothesis was found to be true in *N. caninum *but not in *C. parvum *(*p-values *of 0.010 and 0.960 respectively). The TRP-1 putative binding site identified by Van Poppel and colleagues upstream of *T. gondii *ribosomal genes [24] is also associated with *N. caninum *ribosomal genesbut not *C. parvum *ones (*p-values *of 1.7*10^-19 ^and 0.202). The sequence logo of the TRP-1 site is 'starred' with yellow in Figure 6.

These results enable us to begin assembling a model describing the regulation of ribosomal genes in apicomplexans (Figure 6). In most *Plasmodium *species, *Theileria *and *Babesia *these genes are possibly regulated by one of six candidate regulators mentioned earlier and presented in Additional File 2 (or their respective orthologs in the appropriate species) and the G-box binding site originally identified in *P. falciparum*. In *T. gondii *and the related parasite *N. caninum *these same genes are regulated by a factor similar to the *P. falciparum *AP2 transcription factor PF14_0633 and the site originally associated with *P. falciparum *sporozoite genes together with the TRP-1 site identified by Van Poppel and colleagues [24].

## Discussion

In this work we take advantage of the availability of eleven apicomplexan genomes to examine the conservation of transcriptional regulons in parasites. We compared the conservation profiles of predicted *Plasmodium *transcription factors in disparate apicomplexans to obtain a regulator-centric view of differences in regulons. We also focus on regulon systems associated with four *Plasmodium *binding sites and compare and contrast the conservation of these regulons within the *Plasmodium *genus and in other apicomplexans. Overall, we note large-scale differences in the regulation of regulons as determined by different sets of putative transcription factors in these species. Additionally, in our analysis of binding sites, we note that even when a site is conserved in a particular species, it may be rewired to control a new regulon.

### Conservation and divergence of transcription factors

About three-fifths of *Plasmodium falciparum *predicted transcription factors are conserved in at least one of the other non-*Plasmodium *apicomplexans considered. Additionally, when orthologs of predicted transcription factors from other apicomplexans were sought in *P. falciparum *we noted that generally the further away evolutionarily a species was from *P. falciparum *the smaller the number of detected orthologs. This suggests that over large evolutionary distances the regulators that direct regulons are generally not conserved.

*P. falciparum *transcription factors that have orthologs in non-*Plasmodium *apicomplexans raise the interesting possibility that some transcription factors may be broadly conserved across apicomplexans. These factors may be involved in ancient apicomplexan transcriptional networks. Such factors include TATA-binding proteins and CAAT-box binding transcription factors which are well known general transcription factors, some of which have orthologs in species outside the apicomplexan phylum. These broadly conserved transcription factors also contain Myb, C2H2 zinc finger and AP2 transcription factors which may be involved in core processes a little more specialized than those regulated by TATA-binding and CAAT-binding transcription factors.

### Conservation of the cell invasion and ookinete regulons

Analysis of the *Plasmodium falciparum *cell invasion and *Plasmodium berghei *ookinete transcription factor binding sites suggests that their associated regulons are conserved across *Plasmodium *species. Interestingly, the cell invasion site was observed to be enriched upstream of *Theileria annulata *cell invasion genes as well but not those of the related parasites *Theileria parva *and *Babesia bovis*.

The transcription factor associated with the cell invasion site is conserved in all the *Plasmodium *species and all the piroplasms considered. It is possible that the cell invasion regulon has diverged in *T. parva *and *B. babesia *and the conserved transcription factor has been rewired to serve other regulatory purposes. Alternatively, the lack of enrichment of the cell invasion site in *T. parva *and *B. bovis *may reflect the low number of piroplasm genes with orthologs to known *Plasmodium falciparum *cell invasion genes (Table S1 in Additional File 3). Efforts to better annotate piroplasm genes will likely provide more comprehensive lists of invasion genes specific to these organisms. It will be interesting to determine whether these species-specific invasion genes are also under the control of the *P. falciparum *invasion binding site or another binding site altogether.

The cell invasion binding site, which we demonstrated was conserved in all *Plasmodium *species, is utilized in additional exclusive ways in *P. falciparum*. A combination of affinity purification and mass spectrometry has been used by Voss and colleagues to validate the cell invasion binding site, which they refer to as SPE2, in the upstream regions of a subset of *P. falciparum var *genes [21]. *Var *genes are important parts of the pathways that *P. falciparum *uses to evade the human immune system. *Var *genes are specific to *P. falciparum *so the presence of the cell invasion site in *var *gene upstream regions is a species-specific innovation.

In contrast to the cell invasion binding site, the ookinete site is conserved only in *Plasmodium *species (Figure 4). In this case, the factor is conserved across all *Plasmodium *species, with no conservation observed in any of the piroplasms.

### Transcriptional regulation of ribosomal genes in apicomplexans

*Plasmodium *species and the piroplasms share a ribosomal gene regulon. In addition to controlling ribosomal genes, the binding site associated with this regulon is suspected to control other trophozoite-specific metabolic processes [25]. The observation that *Theileria *species possess a binding site similar to the G-box binding site identified in *P. falciparum *was first made by Guo and Silva [8]. We show that upstream of ribosomal genes, the G-box is conserved across five *Plasmodium *species and three piroplasms but not in the three eucoccidioridian species considered.

The regulation of ribosomal genes illustrates the complexity of transcriptional rewiring in apicomplexans (Figure 6). The G-box was conserved in *Plasmodium *species and piroplasms but not in eucoccidioridians. Upstream regions of ribosomal genes in the eucoccidioridians, *T. gondii *and *N. caninum*, are enriched for the binding site that was originally associated with *Plasmodium *sporozoite genes and a second site, TRP-1, that is not enriched upstream of the orthologous genes in the other apicomplexans. Interestingly, none of the three aforementioned ribosomal gene binding sites are enriched upstream of *C. parvum's *ribosomal genes implying the existence of disparate but yet unknown transcription factor binding sites for this parasite's ribosomal genes.

Divergence of the binding sites governing ribosomal gene regulation despite the core and conserved nature of these genes in species is not unprecedented. The upstream regions of *Saccharomyces cerevisiae *ribosomal genes are enriched for the RAP1 binding site but this particular site is not enriched in the upstream regions of the corresponding genes in *Schizosaccharomyces pombe *[26]. While both *S. cerevisiae *and *Schizosaccharomyces pombe *have copies of the RAP1 transcription factor, the *S. cerevisiae *copy has a transactivation domain that the *Schizosaccharomyces pombe *copy lacks suggesting that the factor and its corresponding site are rewired together. Additionally, the upstream regions of mitochondrial ribosomal genes in yeast species exhibiting a preference for aerobic respiration are enriched for the RGE transcription factor binding site while no enrichment is seen upstream of the same genes in yeast species with a preference for anaerobic respiration [27].

### Evolution of a sporozoite regulon via rewiring of binding sites

A phylogenetic order-specific system is involved in sporozoite gene regulation in *Plasmodium *species. This suggests that systems governing the control of sporozoite gene expression differ between *Plasmodium *species on one hand and the rest of the apicomplexans on the other.

The binding site originally associated with the *Plasmodium *sporozoite regulon described above has different roles in other apicomplexans suggesting that it illustrates a case where a system evolves by rewiring of sites in different species. Specifically, the site is enriched upstream of ribosomal genes in the two eucoccidioridians *T. gondii *and *N. caninum*. Additionally, others have suggested that the transcription factor associated with the site is in fact conserved in all apicomplexans and also that this factor is associated with different sets of genes in *Plasmodium falciparum *and *Cryptosporidium parvum *[13]. It is worth noting that this factor is likely multifunctional even in *Plasmodium *species, as it has been demonstrated to be active in *Plasmodium *red blood cell and sporozoite stages [13,28].

### A role for adaptive evolution in the conservation patterns of transcriptional regulons in apicomplexans?

Differences in the conservation of putative transcription factors and known binding sites suggest that regulons have diverged between apicomplexan species. A natural question that arises from the results of this study is whether the observed differences are the result of neutral or adaptive evolution.

Under the neutral evolutionary scenario, over evolutionary time, orthologous transcription factors have accumulated so many mutations, albeit neutral ones, that though their functions may remain the same they are no longer identifiable as orthologs by computational means.

While experimental evidence for adaptive changes in transcription factors is not available for apicomplexans, there is computational evidence to suggest that such adaptation is highly likely. In higher eukaryotes, coding regions of transcription factors have been observed to diverge more quickly than those of genes involved in housekeeping processes like metabolism [29]. In a study of five *Plasmodium *species we noted that *Plasmodium *transcription factors and parasite lifestyle-associated genes might behave similarly as they have less constrained coding regions (higher dN/dS or rates of nonsynonymous substitutions per nonsynonymous site to synonymous substitutions per synonymous site) than other groups of genes [30].

The cell invasion, ookinete and sporozoite binding sites all have some *Plasmodium*-specific conservation patterns. Though instances of these sites are present in all the apicomplexan species considered, they all have sets of genes involved in a biological process or lifecycle stage whose upstream regions are enriched for these sites in *Plasmodium *species only (in the case of the cell invasion site we see association in one of the three piroplasms as well). The ribosomal genes have a specific binding site for *Plasmodium *species and piroplasms and another set of binding sites for *T. gondii *and *N. caninum*. Such changes in regulons are not unprecedented. In a similar study of yeast species separated by 420-600 million years of evolution only one out of forty-two regulons was conserved across fourteen species considered. Additionally, more distantly related yeast species shared fewer binding sites [31].

The lack of conservation of binding sites in all apicomplexans could result from neutral evolution - over 700-900 millions years of apicomplexan evolution a remarkable amount of drift is expected in the upstream regions of distantly related parasites.

Alternatively, these regulon systems might be evolving adaptively in response to genus-related lifestyle and environmental differences. This is possible given that all the binding sites considered were correlated with specific genera or groups of genera within the phylum. However, further work is necessary to distinguish between these two scenarios. Probabilistic methods exist for examining the types of selection, if any, on binding sites within closely related organisms - yeast species (20 million years of separation) and fly species (10 million years of separation) - however these methods require high quality alignments [32,33] which are not easy to generate for the distantly related apicomplexans in our study.

## Conclusions

In this work, we demonstrate the power of comparative genomics in shedding light on transcriptional regulation in non-model genomes with little prior knowledge. We integrate eleven apicomplexan genomes with observations from high-throughput regulation studies and highlight different modes of transcriptional regulon modification. While transcription factors are underrepresented in these parasites it appears that these species use the few factors they have in phylogenetic order-specific ways. As more annotation, expression data and results from experimental studies of transcription factors in these parasites become available it will be possible to develop a more global perspective of transcriptional differences, and their functional consequences if any, in these important parasites.

## Methods

### Orthology

Orthologs were predicted via two methods. The first method is referred to as the reciprocal best BLAST hits (RBH) approach. Under the RBH framework, orthologs were determined by identifying reciprocal best BLAST hits between various apicomplexans. An E-value cutoff of 10^-5 ^was utilized. OrthoMCL implements a more rigorous approach for the identification of orthologs. OrthoMCL first identifies reciprocal best BLAST hits between species and reciprocal 'better' BLAST hits within species. It utilizes an E-value cutoff of 10^-5^. Markov Clustering (MCL) is then used to better define the orthology and paralogy relationships between proteins from the resulting graph of BLAST E-values [17,34].

Orthologs were primarily obtained during the course of this work from two runs (versions 2.1 and 3.0) of OrthoMCL-DB [17] kindly provided by Deborah Pinney. OrthoMCL version 2.1 covering 89 genomes was used for the identification of orthologous apicomplexan upstream regions. The *Babesia bovis *genome is currently not included in OrthoMCL-DB so *P. falciparum *- *B. bovis *orthologs were obtained from a separate run of the OrthoMCL algorithm kindly provided by Kuo and colleagues [16]. For more comprehensive analysis of orthologous transcription factors, OrthoMCL version 3.0 covering 128 genomes was used for the transcription factor orthology analysis. In one case, OrthoMCL 3.0 did not identify any haemosporidian orthologs for a *P. falciparum *factor (MAL8P1.153), but examination of syntenic regions revealed that orthologs do in fact exist in the two other primate malaria parasites. Apicomplexan orthologs for the 45 *P. falciparum *predicted transcription factors are presented in Additional File 5.

### Identification of putative transcription factors

Known DNA-binding domains [3,35] were obtained from version 23.0 of the Protein families database (Pfam) [36]. Hidden Markov Model representations of these domains were scanned against apicomplexan proteomes to identify putative transcription factors. A protein is defined as containing a domain if its E-value exceeds the domain-specific Pfam-defined gathering threshold. The gathering threshold of a specific Pfam Markov Model is the sequence-based cutoff at which the Pfam curators "gather" sequences to include in that Markov Model. 45 putative transcription factors were identified in *P. falciparum*. The predicted transcription factors in each of the apicomplexans are presented in Additional File 1.

### Transcription factor binding sites

The G-box position weight matrix (PWM) was constructed from the original sequences discovered by Militello and colleagues [22]. The sporozoite and cell invasion PWMs were constructed from the sequences used to generate the *P. falciparum *versions of the respective motifs from earlier work from our lab [5]. In their paper [23], Yuda and colleagues did not provide the raw individual ookinete binding sites necessary for constructing a PWM. Consequently, we ran the motif finder MEME (version 4.2.0) [37] on the upstream regions of the ookinete genes reported by Yuda et al. to identify the ookinete PWM.

### Expected regulons

The sporozoite regulon consisted of a set of genes maximally expressed in sporozoites as determined in previous publications [30,38]. The ribosomal regulon consisted of a set of genes annotated as being part of the ribosome (GO:0005840) using Gene Ontology annotation obtained from PlasmoDB on 10/19/2008. As the Gene Ontology has very few *P. falciparum *genes annotated as being involved in cell invasion, the cell invasion regulon consisted of genes obtained from an integrated clustering of Gene Ontology terms and expression data [39]. The ookinete regulon consisted of the set of genes found to be maximally expressed in ookinetes extracted from the study by Vontas and colleagues [40].

### Assessment of binding site conservation

The best scoring instance of a particular binding site in the region 2000 bp upstream of each gene (or up until the nearest upstream gene if that was closer) in a genome of interest was identified using PWM_SCAN with the 'stage 2' option which uses the percentile of the observed score between the minimum and maximum achievable by the matrix as a hit score [41]. The average length of intergenic regions in the five *Plasmodium *species considered is 1476.58 bp (computed from data in [42,43]). By limiting our analyses to a maximum upstream length of 2000 bp, on average, we capture most of the entire intergenic regions in these species and give some extra allowance for longer upstream regions. Given that most of the binding sites were originally discovered in *Plasmodium *species and also for consistency we anchored the maximum lengths of upstream regions in non-*Plasmodium *species at 2000 bp as well.

To identify a score cutoff for high scoring hits in a particular genome, the position weight matrix (PWM) of each site was permuted 100 times to obtain 100 random copies of that site, each with the same base composition as the original site. These 100 permuted sites were also used to scan the upstream genomic regions. Two distributions were then created, one consisting of the scores of the actual site and the other, a background distribution, consisting of the scores of the all the permuted sites. The score at the top one percentile of distinct scores in the background distribution was used as a cutoff for high confidence hits of the actual site. Next, the overlap between genes with high confidence hits of the site in their upstream regions and the set of genes expected to be regulated by the site was assessed by estimating the probability of obtaining the observed amount of overlap (enrichment) by random chance using the hypergeometric distribution. The above procedure was repeated in each genome of interest for the particular site (using the same permuted instances of the site) and the *p-values *from the hypergeometric distribution were compared across species to assess conservation. The procedure is explained pictorially in Figure 2.

Hypergeometric statistics were computed using the *phyper *function in the R statistical language. In computing the hypergeometric statistics we used the total number of predicted genes rather than the total number of available upstream regions as the total set size. Genes on short contigs may not have upstream regions or may have unrealistically short upstream regions. By utilizing the total number of genes as the total set size in the hypergeometric computation we are being overly conservative and improved annotation resulting in new upstream regions may improve *p-values*. We exclude upstream regions less than 10 bp - see Table 1 for total numbers of available upstream regions.

**Table 1:** Apicomplexan genomes utilized in this paper

**Abbr**.	Species	Version	Genome Status	Source	Primary reference	Number of genes	Number of available upstream regions
*Cp*	*Cryptosporidium parvum*	02/23/2007	13X	CryptoDB	[44]	3886	3875

*Tg*	*Toxoplasma gondii*	07/23/2008		ToxoDB		8155	8120

*Nc*	*Neospora caninum*	08/05/2008		ToxoDB		5761	5746

*Bb*	*Babesia bovis*	04/10/2008	8X	GenBank	[45]	3671	3657

*Tp*	*Theileria parva*	07/30/2005	Complete	GenBank	[46]	4035	4025

*Ta*	*Theileria annulata*	07/29/2005	8X	GeneDB	[4]	3793	3786

*Pf*	*Plasmodium falciparum*	06/28/2007	Complete	PlasmoDB	[2]	5460	5456

*Pk*	*Plasmodium knowlesi*	02/22/2007	8X	GeneDB	[47]	5161	4762

*Pv*	*Plasmodium vivax*	09/11/2007	Complete	GenBank	[5]	5390	5342

*Pb*	*Plasmodium berghei*	02/27/2006	4X	PlasmoDB	[43]	12365	10238

*Py*	*Plasmodium yoelii yoelii*	09/10/2002	5X	PlasmoDB	[48]	8075	7603

Overlap (enrichment) statistics for the cell invasion, ribosomal/G-box and sporozoite sites are given in Tables S1, S2 and S3 respectively (all in Additional File 3). We also assessed the ribosomal genes for enrichment with the sporozoite site and the TRP-1 site discovered by Van Poppel and colleagues [24]. The overlap statistics for these two sets of analyses are in Tables S4 and S5 respectively in Additional File 3.

## Authors' contributions

KE: conceived and designed analyses, performed analyses and drafted the manuscript. CJS: conceived and designed analyses and assisted in drafting the manuscript. All authors read and approved the final manuscript.

## Supplementary Material

Additional file 1 **Putative transcription factors in apicomplexan proteomes**. This file contains Pfam-predicted transcription factors in the apicomplexan species considered in this paper. It has multiple sheets. Each sheet has two columns - the first of which specifies a DNA-binding domain and the second a protein predicted to contain that domain. The sheets are labelled according to the species abbreviations described in Table 1. Click here for file

Additional file 2 **Putative transcription factors with similar orthology profiles to the G-box binding site**. This file contains the putative transcription factors whose orthology profiles are similar to that of the G-box. Their DNA-binding domains and correlation to the average expression profile of the putative G-box associated ribosomal genes (Additional File 4) are also given. Click here for file

Additional file 3 **Data used for computing binding site enrichment statistics**. Contains labelled supplementary tables presenting the data used to compute the binding site enrichment *p-values *for the various regulons. Click here for file

Additional file 4 **Ribosomal genes with high confidence G-box hits in their 2000 bp upstream regions**. This file contains the ribosomal genes with high confidence G-box hits in their 2000 bp upstream regions (data used to construct Figure 5). The file has two columns - the first specifies gene names and the second states gene annotation obtained from PlasmoDB. Click here for file

Additional file 5 **Orthologs of the putative *P. falciparum *transcription factors**. This file contains orthologs (one species per column) of each the putative *P. falciparum *transcription factors. 'NA' implies that an ortholog was not detected in a particular species. Columns are labelled according to the species abbreviations described in Table 1. The two MAL8P1.153 orthologs identified by syntenic examination are highlighted in red. Click here for file
