# Supplementary material for: Conservation and divergence of known apicomplexan transcriptional regulons
Source: BMC Genomics. 2010 Mar 3;11:147. doi: 10.1186/1471-2164-11-147 (PMC2841118; doi:10.1186/1471-2164-11-147)
Supplement: Additional file 3 — Data used for computing binding site enrichment statistics. Contains labelled supplementary tables presenting the data used to compute the binding site enrichment p-values for the various regulons. [file 1471-2164-11-147-S3.PDF]

Table S1 - Statistics of the overlap between the expected cell invasion regulon and the upstream regions with PWM hits above the threshold

Species	Regulon size	Upstream regions in genome with hits above threshold	Overlap	*p-value*
C. parvum	7	120	0	1
T. gondii	14	2750	4	0.749
N. caninum	12	2491	3	0.946
B. bovis	11	331	2	0.261
T. parva	13	75	1	0.217
T. annulata	11	85	2	0.024
P. falciparum	87	348	16	$9.6*10^{-5}$
P. knowlesi	50	2005	31	0.0007
P. vivax	46	2408	31	$1.5*10^{-3}$
P. berghei	41	427	4	0.052
P. yoelii	37	387	7	0.002

Table S2 - Statistics of the overlap between the expected ribosomal/G-box regulon and the upstream regions with PWM hits above the threshold

Species	Regulon size	Upstream regions in genome with hits above threshold	Overlap	*p-value*
C. parvum	81	73	3	0.194
T. gondii	119	1500	23	0.433
N. caninum	102	1553	23	0.871
B. bovis	104	197	12	0.009
T. parva	120	76	11	$1.1*10^{-5}$
T. annulata	119	70	9	0.0003
P. falciparum	155	151	11	0.004
P. knowlesi	139	1516	55	0.006
P. vivax	140	2475	80	0.005
P. berghei	109	294	7	0.015
P. yoelii	124	248	7	0.087

Table S3 - Statistics of the overlap between the expected sporozoite regulon and the upstream regions with PWM hits above the threshold

**Species**	**Regulon size**	**Upstream regions in genome with hits above threshold**	**Overlap**	***p-value***
C. parvum	96	961	22	0.699
T. gondii	154	1676	33	0.425
N. caninum	142	1170	28	0.604
B. bovis	123	12	0	1
T. parva	139	27	1	0.613
T. annulata	136	24	1	0.585
P. falciparum	450	373	66	$6.9*10^{-10}$
P. knowlesi	311	383	44	$1.6*10^{-5}$
P. vivax	308	472	47	$8.6*10^{-5}$
P. berghei	246	808	30	0.0007
P. yoelii	285	867	53	$3.8*10^{-5}$

Table S4 - Statistics of the overlap between the expected ribosomal regulon and the upstream regions with hits for the sporozoite PWM that are above the threshold

**Species**	**Regulon size**	**Upstream regions in genome with hits above threshold**	**Overlap**	***p-value***
C. parvum	81	961	14	0.960
T. gondii	119	1676	33	0.036
N. caninum	102	1170	31	0.010
B. bovis	104	12	0	1
T. parva	120	27	1	0.559
T. annulata	119	24	0	1
P. falciparum	155	373	4	0.995
P. knowlesi	139	383	4	0.994
P. vivax	140	472	8	0.933
P. berghei	109	808	13	0.025
P. yoelii	124	867	15	0.352

Table S5 - Statistics of the overlap between the expected ribosomal regulon and the upstream regions with hits for the TRP-1 PWM that are above the threshold

Species	Regulon size	Upstream regions in genome with hits above threshold	Overlap	*p-value*
C. parvum	81	40	2	0.202
T. gondii	119	277	27	$1.6*10^{-15}$
N. caninum	102	218	30	$1.7*10^{-19}$
B. bovis	104	22	0	1
T. parva	120	15	1	0.365
T. annulata	119	31	0	1.000
P. falciparum	155	60	1	0.824
P. knowlesi	139	21	0	1
P. vivax	140	23	1	0.455
P. berghei	109	96	1	0.574
P. yoelii	124	89	2	0.398

Table S6 - Statistics of the overlap between the expected ribosomal regulon and the upstream regions with hits for the cell invasion PWM that are above the threshold

Species	Regulon size	Upstream regions in genome with hits above threshold	Overlap	*p-value*
C. parvum	81	120	1	0.923
T. gondii	119	2750	30	0.983
N. caninum	102	2491	48	0.246
B. bovis	104	331	5	0.965
T. parva	120	75	1	0.898
T. annulata	119	85	0	1.000
P. falciparum	155	348	7	0.874
P. knowlesi	139	2005	33	1.000
P. vivax	140	2408	55	0.918
P. berghei	109	427	4	0.523
P. yoelii	124	387	3	0.941
